# Clinical Evaluation of 3D High Resolution Late Enhancement using Image-Based Navigation

**DOI:** 10.1186/1532-429X-18-S1-P310

**Published:** 2016-01-27

**Authors:** Konstantinos Bratis, Chrysanthos Grigoratos, Marcus Henningsson, Matteo Dell'Omodarme, Rene M Botnar, Eike Nagel

**Affiliations:** 1 King's College London, London, United Kingdom; 2 Physics, University of Pisa, Pisa, Italy

## Objective

To prospectively assess the diagnostic performance of high resolution image-navigated 3-dimensional late gadolinium enhancement (iNAV-3D LGE) magnetic resonance imaging (CMR) for the detection of myocardial necrosis in a routine clinical setting.

## Background

iNAV-3D LGE is a novel CMR technique which allows for direct respiratory motion correction of the heart. However, its performance in real-life clinical scenarios has not yet been established.

## Methods

23 consecutive patients referred for CMR examination including scar imaging were prospectively enrolled. Gadolinium enhanced (0.20 mmol/kg Gd-DTPA) navigated high resolution (2 mm3 isotropic) 3D T1-weighted gradient-echo inversion recovery sequence using image-based navigation in comparison with a conventional two-dimensional (2D LGE) sequence were performed in random order by using a 1.5-T clinical MR imaging system. Images were assessed qualitatively with regard to the detection of global and segmental LGE and transmurality. Additional subjective image quality assessement including image quality, mean LGE signal intensity and LGE-myocardial/ blood pool sharpness on a 4-point scale was performed.

## Results

**Figure 1:**
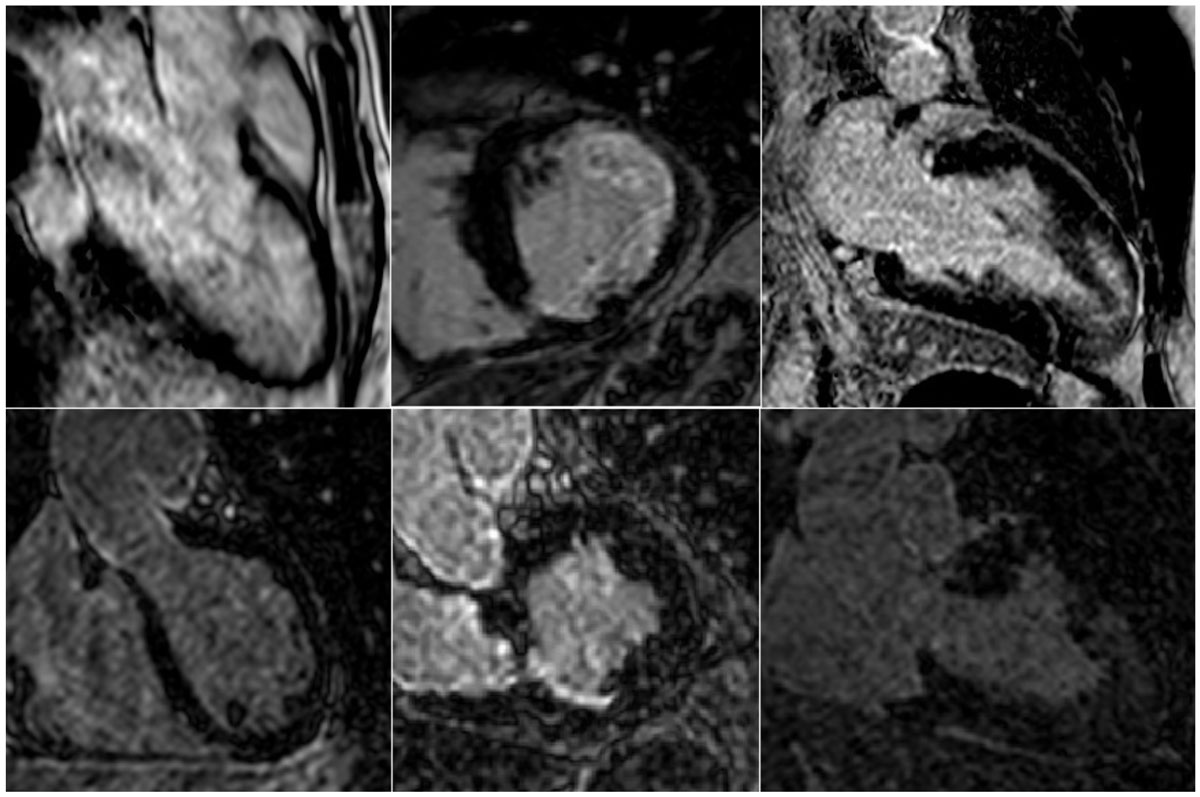
**Selected matched images of 2D (upper row) and iNav-3D (lower row) LGE in a patient with dilated cardiomyopathy (left), ischaemic heart disease (mid) and hypertrophic cardiomyopathy (right)**. LGE: Late Gadolinium Enhancement

Interpretable images were obtained in all 2D-LGE and in 22/ 23 iNAV-3D LGE exams, resulting in a total of 22 complete sequence datasets and 352 segments. LGE was detected in 5 patients with ischemic pattern, in 8 with non-ischaemic pattern, while it was absent in 9 cases. (Figure 1) There were no significant differences between 2D and 3D data sets with regard to global and segmental LGE detection (p, 0.13 and p, 0.28, respectively) and transmural extension (p, 0.84). Agreement regarding image quality was good, but 2D LGE presented higher LGE mean signal intensity. (Table 1) The average acquisition time for iNav-3D was from 4-6 minutes. Results were similar indepentently of the order in which the sequences were performed.

**Table 1:** Comparison of the main diagnostic and quality assessment parameters for 2D and iNav3D LGE.

DIAGNOSTIC PERFORMANCE						
Global LGE detection (p, 0.13)						
2D (n = 22)	77% (17)					
iNav3D (n = 22)	59% (13)					
Segmental LGE detection (p, 0.28)						
Number of segments 0 1 2 3 4 5						
2D (n = 352)	75.9% (267)	10.2% (36)	3.7% (13)	7.7% (27)	2% (7)	0.6% (2)
iNav 3D (n = 352)	78.1% (275)	8.5% (30)	2% (7)	7.4% (26)	3.4% (12)	0.6% (2)
IMAGE QUALITY						
Mean (SD)	2D LGE		iNav 3D LGE		p value	
Quality (n = 22)	3 (0.9)		3.4 (0.8)		0.34	
LGE- blood pool sharpness (n = 13)	2.8 (0.8)		2.8 (0.8)		0.48	
Myocardium- LGE sharpness (n = 13)	3 (0.8)		3 (0.8)		0.48	

LGE: Late Gadolinium enhancement, n: number, segmental LGE detection: 0= no LGE, 1= ischaemic, 2= patchy, 3= subepicardial, 4= mid wall, 5= RV insertion points.

## Conclusions

In this study, imaging performance and quality scores of iNAV-3D LGE images were comparable to those of 2D LGE in a prospective clinical setting. iNAV-3D LGE may potentially offer a reliable alternative for high quality scar imaging.

